# Incidence of clinically relevant psychiatric symptoms during glioblastoma treatment: an exploratory study

**DOI:** 10.1007/s11060-023-04326-2

**Published:** 2023-05-10

**Authors:** L. K. P. Regli, S. M. H. Huijs, R. C. O. S. Pasmans, C. Leue, J. B. Dijkstra, D. B. P. Eekers, K. E. Hovinga, M. H. M. E. Anten, A. Hoeben, M. P. G. Broen

**Affiliations:** 1 Department of Neurology, Maastricht University Medical Center, P.O. Box 5800, 6202 AZ Maastricht, The Netherlands; 2 Department of Neurology, Zuyderland Medical Center, Heerlen, The Netherlands; 3 Department Psychiatry, Maastricht University Medical Center, Maastricht, The Netherlands; 4 Department of Medical Psychology, Maastricht University Medical Center, Maastricht, The Netherlands; 5 Department of Radiation Oncology (Maastro), GROW School for Oncology and Reproduction, Maastricht University Medical Centre+, Maastricht, The Netherlands; 6 Department of Neurosurgery, Maastricht UMC+, Maastricht, The Netherlands; 7 GROW-School for Oncology and Reproduction, Maastricht University, Maastricht, The Netherlands; 8 Department of Medical Oncology, Maastricht UMC+, Maastricht, The Netherlands; 9 MHeNS, School for Mental Health and Neuroscience, Maastricht University, Maastricht, The Netherlands

**Keywords:** Glioblastoma, Psychiatric symptoms, Chemoradiation, Psychosis, Temozolomide

## Abstract

**Purpose:**

In addition to neurological symptoms glioblastoma (GBM) patients can experience psychiatric complaints, which are often hard to recognize and difficult to treat. Research on psychiatric symptoms during glioblastoma treatment is limited, but can have significant impact on quality of life, treatment processes and even survival. The aim of this study is to explore the incidence of clinically relevant psychiatric symptoms, during glioblastoma treatment and active surveillance.

**Methods:**

Medical records of 302 GBM patients were reviewed from diagnostic surgery until discontinuation of treatment or active surveillance. Clinical relevance was defined as psychiatric symptoms that interfered with the oncological treatment and required referral to a psychiatrist. “Referred” versus “non-referred” GBM patients were compared using the Pearson Chi-Square test, Fisher’s Exact Test or Mann Whitney-U test.

**Results:**

Psychiatric symptoms occurred in 11.5% of patients during glioblastoma treatment or active surveillance, most often mood or behavioral symptoms, followed by psychotic symptoms. Referral occurred mainly during concomitant chemoradiation or adjuvant chemotherapy (64.3%). In 28.6% of patients psychiatric symptoms were thought to be attributive to medication. Treatment was discontinued in 17.9% of patients and temporarily interrupted in 3.6%. Possible risk factors included male gender, history of psychiatric disorder, postoperative delirium, non-frontal tumor location, anti-epileptic drug use at baseline and corticosteroid initiation during treatment.

**Conclusion:**

The found incidence of 11.5% and the high number of patients discontinuing treatment due to psychiatric symptoms justify more research in this, to date, understudied topic in scientific literature. Further prospective studies are needed to identify risk factors and unravel possible effects on survival.

**Supplementary Information:**

The online version contains supplementary material available at 10.1007/s11060-023-04326-2.

## Introduction

Glioblastoma (GBM) is defined as a grade IV, isocitrate dehydrogenase 1/2 gene (IDH1/2) wild type astrocytic glioma with no mutations in histone H3 genes and is characterized by microvascular proliferation, necrosis and/or specific prognostic molecular features. [1] This primary brain tumor is rare and has an annual incidence of 3–5 per 100.000 people with a median age of onset of 65 years. [2, 3] To this day no cure has been found and treatment is only life-prolonging. Standard treatment includes maximal safe resection or biopsy followed by a treatment schedule comprising radiotherapy and chemotherapy (temozolomide). [1, 4] However, despite this extensive multimodal treatment schedule, the median survival is limited to only 14–16 months. [2].

In GBM, both the disease and its treatment have a direct effect on brain functioning. Patients commonly experience neurological, cognitive or psychiatric impairment during diagnosis and treatment. [5] A lot of research and attention is given to neurological and cognitive deterioration in GBM patients, however broader psychiatric symptoms are often undetected and seldom primary topic of research interest. This is despite the fact that the reported incidence of psychiatric symptoms in primary brain tumors vary between 50 and 78% [6], comprising a wide variety of disorders such as mania, psychosis, anxiety disorders or personality change. One possible explanation for the lack of research on psychiatric disturbances is that they are difficult to recognize and hard to treat. They often become more apparent as the disease progresses and this can affect patient’s ability to engage in shared decision making and impairs their quality of life. [5] Although some researchers attempted to investigate psychiatric burden in patients with brain tumors, none specifically focuses on GBM patients. [3, 5, 7] In addition, these studies mainly reported psychiatric symptoms as presenting symptom of a brain tumor and did not describe their primary occurrence during tumor treatment or their interference with the oncological treatment. [8–13].

Because of the detrimental effects of psychiatric symptoms on patients’ quality of life [5, 13] we hypothesize that a better understanding of the incidence of psychiatric symptoms during GBM treatment and follow up may lead to better patient education, earlier recognition and therefore more efficient upfront treatment. This may improve patients’ quality of life and allow a fully completed oncological treatment, ultimately optimizing survival. With this aim, we retrospectively explored the incidence of clinically relevant psychiatric symptoms, defined as symptoms interfering with the oncological treatment during therapy or active surveillance in a multicenter cohort of GBM patients.

## Methods

### Patient selection

A Dutch multicenter retrospective study was performed at the Maastricht University Medical Center (MUMC +), Maastro Radiotherapy Institute (Maastro) and Zuyderland Medical Center (ZMC). In total 302 newly diagnosed GBM patients, diagnosed or treated in MUMC + , Maastro and/or ZMC between 2011 and 2020, were reviewed. Inclusion criteria for this study were adult (> 18 years) GBM cases with full information on IDH1/2, 1p/19q copy number status and methylguanine DNA Methyltransferase (MGMT) methylation status available.

Patient characteristics and clinical data were collected from electronical medical records. The following characteristics and variables were used for this study: gender, age at diagnosis, ECOG Performance Status at baseline (score 0–4), type of surgery (biopsy or resection), tumor location, use of dexamethasone at baseline, use of anti-epileptic drugs at baseline, use of antipsychotic drugs at baseline, history of psychiatric disorder, symptoms at first presentation, initial GBM treatment (type of treatment, duration of treatment, early discontinuation of Stupp/Elderly treatment [4]) and overall survival (OS). The reason for discontinuation of treatment (e.g., disease progression, medication toxicity, psychiatric disturbances) were extracted from records of multidisciplinary tumor board meetings. OS was defined as time from date of diagnosis (set as date of diagnostic surgery) to date of death or date of last follow up for patients still alive.

### Assessment of clinically relevant psychiatric symptoms

The primary outcome was the incidence of clinically relevant psychiatric symptoms during GBM treatment or active surveillance. Clinically relevance was defined as psychiatric symptoms that required a referral to a psychiatrist, according to the clinical judgement of the treating physician, since symptoms were interfering with the optimal oncological treatment course. This could either be during radio- or chemotherapy or during active surveillance after completion of therapy. Only new symptoms were scored. Symptoms that were already present at the initial tumor presentation were only scored if these symptoms clearly worsened during treatment or surveillance and therefore required a first psychiatric consultation. Medical records were analyzed from diagnostic surgery until the discontinuation of treatment or surveillance. Treatment discontinuation was defined as stop of treatment or surveillance for medical reasons (switch treatment plan to best supportive care (BSC)), stop surveillance because of clinical deterioration or stop at patients request or sudden death. The first weeks after surgery were separately analysed to distinguish more transient psychiatric symptoms due to direct influences of surgery or anaesthetics from more prolonged psychiatric symptoms that could develop during treatment or follow up. This period was defined as ‘postoperative period’ and encompasses the time between diagnostic surgery until the first follow-up appointment at the outpatient clinic at which pathology results and subsequent treatment plan were discussed.

Collected variables were (a) descriptive psychiatric symptoms as mentioned by patients or caregivers during clinic visits and reported in medical records by the treating physician, (b) consultations with a psychiatrist, including their evaluation, final diagnosis and treatment plan, (c) consultations with an external psychiatrist and (d) the occurrence of a postoperative delirium. Postoperative delirium was defined as an episode of confusion or agitation for which treatment was started, or if a delirium was clearly stated as a diagnosis in the medical file. In accordance with common general practice descriptive symptoms and psychiatric diagnoses were divided into six categories: cognitive symptoms, psychotic symptoms, mood disturbances, symptoms of anxiety, behavioral symptoms or changed habit and somatic symptoms, which remained medical unexplained. In an attempt to discriminate cognitive symptoms from primary psychiatric symptoms, we categorized symptoms as ‘cognitive’ when direct brain damage was thought to be the most likely cause, for example memory problems due to a tumor in the frontal or temporal lobe. Otherwise, we classified the symptoms in one of the 5 other categories.

### Statistical analysis

Statistical analysis was performed using SPSS version 27.0 (IBM Corporation, Armonk, NY, USA). A P-value of < 0.05 was considered statistically significant. Descriptive statistics were calculated for all patients. Incidence was calculated for all reported symptoms during outpatient clinic visits, as well as for diagnosis made by a psychiatrist. The statistical difference between groups for categorical variables was assessed using the Pearson Chi-Square test or Fisher’s Exact Test (whichever was appropriate according to the number of cases), for continuous variables the Mann–Whitney U test was used. Treatment was reported in a descriptive way. Kaplan–Meier analysis was used to explore differences in overall survival between “referred” and “non-referred” patients.

## Results

### Cohort characteristics

**Fig. 1:**
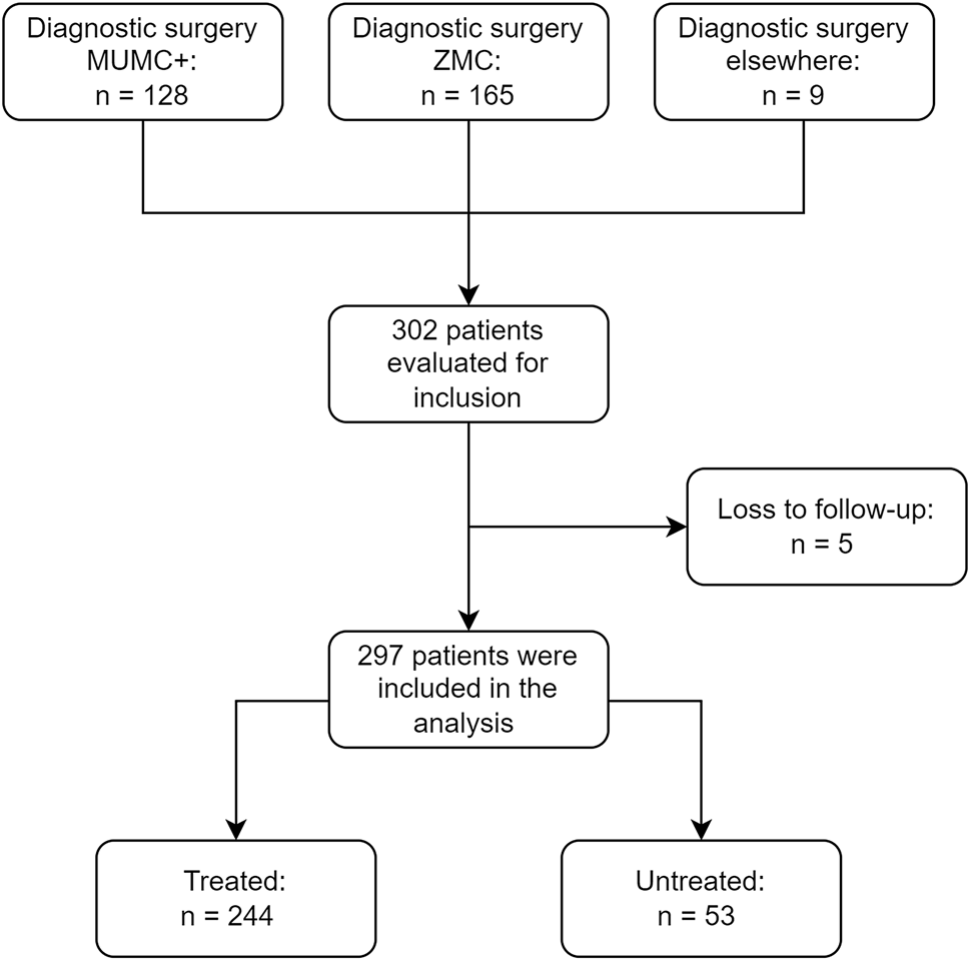
Flowchart of the selection process

In total 302 patients were evaluated for inclusion. Three patients continued treatment in a different hospital and 2 patients had incomplete records regarding treatment initiation, hence in total 5 patients were considered loss to follow up and were not included in the final analysis, see Fig. 1. The final cohort consisted of 297 patients; their characteristics are exhibited in Table 1. At time of analysis 282 patients (94.9%) were deceased, 11 patients (3.7%) still received treatment or were in active surveillance and 4 patients discontinued treatment but were not yet deceased. In total 244 patients (82.2%) received treatment whereas 53 patients (17.8%) were not treated after diagnostic surgery. Reasons for getting no treatment were rapid clinical deterioration (85%) or no initiation of treatment at request of patient and caregiver (15%). The characteristics of the treated patients and the patients who did not receive any treatment are shown in *supplementary table 1.* Treated patients were significantly younger at diagnosis compared to non-treated patients. In addition, they underwent more frequently a resection as diagnostic surgery, had a better ECOG score at diagnosis and, as expected, a longer overall survival time.

**Table 1:** Baseline characteristics

Variables	Study cohort (n = 297)
Gender, n (%)	
Female	108 (36.4)
Male	189 (63.6)
Age at diagnoses, years	
Mean (SD)	64.4 (10.2)
Median (range)	65.7 (26.0–88.0)
MGMT hypermethylation, n (%)	
Yes	123 (41.4)
No	174 (58.6)
Type of surgery, n (%)	
(Partial) resection	146 (49.2)
Biopsy	151 (50.8)
Glioblastoma first line treatment, n (%)	
No treatment	53 (17.8)
Stupp/Elderly* protocol, finished	95 (32.0)
Stupp/Elderly* protocol, not finished	94 (31.6)
Radiotherapy only (6 weeks)	27 (9.1)
Radiotherapy only, not finished	3 (1.0)
Temozolomide only (6 cycles)	2 (0.7)
Temozolomide only, not finished	21 (7.1)
Chemoradiation with 12 cycles adjuvant temozolomide	2 (0.7)
Overall survival (OS), months (SD)	
All patients, mean	11.6 (10.8)
Treated patients, mean	13.6 (10.8)
Non-treated patients, mean	1.9 (1.6)
ECOG score at baseline, n (%)	
0 or 1	209 (70.4)
≥ 2	88 (29.6)
Use of dexamethasone at baseline, n (%)	
Yes	204 (68.7)
No	93 (31.3)

n = number; SD = standard deviation; MGMT = 06-methylguanine-DNA-methyltransferase;

*ECOG* Eastern Cooperative Oncology Group

*Stupp protocol = 6 weeks chemoradiation with 6 cycles adjuvant temozolomide / Elderly protocol = 3 weeks chemoradiation with 6 cycles adjuvant temozolomide

### Postoperative period

In 288/297 (97%) patients, information was available about the postoperative period. Nine patients underwent diagnostic surgery in a different hospital, and therefore no records could be reviewed. During the postoperative period, 11/288 patients died due to rapid clinical deterioration as a result of tumor progression, 3/288 due to a pulmonary infection and 3/288 due to complications of a massive stroke. Of the remaining 271 patients, 18 (6.6%) developed a postoperative delirium. Visual hallucination was the most prominent reported symptom. All patients were using high dose of dexamethasone (> 4 mg daily dose) at that time, according to standard postoperative protocol. The cause of the delirium was most often described as multifactorial, including adverse effects of the steroid. Eleven patients were treated with medication, mainly haloperidol or lorazepam, the remaining 7 patients received non-medical or conservative treatment. In 5/18 patients (27.8%) a psychiatric consultation took place for evaluation and treatment advice, both medical and non-medical. Due to the development of a postoperative delirium, initiation of glioblastoma treatment was delayed in 3 patients for 1 month, 2 weeks and 1 week respectively.

### Reported symptoms during treatment and active surveillance

All 244 treated patients were reviewed. Starting at the first follow up appointment after surgery various symptoms mentioned by the patients and caregivers were reported in medical records. Ninety-one percent of patients mentioned one or more symptoms during at least one hospital visit. Most often fatigue, followed by memory problems, confusion (not further specified), feeling down, coping difficulties and medical unexplained loss of appetite or weight loss. For a complete overview of symptoms and their classification see supplementary Fig. 1 and supplementary table 2.

### Incidence of psychiatric symptoms

Of 244 treated patients, 28 (11.5%) developed clinically relevant psychiatric symptoms and were referred to a psychiatrist during GBM treatment or active surveillance. Twenty-five of the consultations actually took place, 2 patients, referred for hallucinations/delusions and behavioural problems respectively, deteriorated quickly before consultation could take place and 1 patient did not receive a psychiatric evaluation for unknown reasons. Only a small portion (28/222, 12.6%) of patients reporting symptoms during outpatient visits were eventually referred to a psychiatrist, with an exception of patients exhibiting psychotic symptoms. Two patients received care from both an extramural psychiatrist as well as hospital psychiatrist. Most referrals were because of mood symptoms, followed by behavioral symptoms or changed habit and psychotic symptoms. At time of consultation, 22/28 patients (78.6%) were using corticosteroids. Table 2 shows the different diagnosis made after psychiatric evaluation, and if applicable, their treatment plan. In 28.6% of patient’s psychiatric disturbances were thought to be attributive to medication, especially corticosteroids (14.3%), anti-epileptic drugs (10.7%) or both (3.6%). Most referrals took place during active treatment with concomitant chemoradiation or adjuvant temozolomide (64.3%, 18/28). In 5 patients (17.9%) psychiatric symptoms were so severe that glioblastoma treatment was definitive stopped and in 1 patient (3.6%) GBM treatment was temporarily interrupted for 3 weeks.

**Table 2:** Overview of psychiatric evaluations, diagnosis and treatment plans

Reason for referral	Differential diagnosis	Treatment plan
Cognitive symptoms (n = 4)	Multiple cognitive disorders and limited disease insight, incompetent #	No specific psychiatric treatment
	Multifactorial delirium (e.g., medication) #	Medical treatment
	Anxiety and mood disorder, no cognitive disorder	Psychotherapy
	Disorientation and confusion, no cognitive or psychiatric disorder	No specific psychiatric treatment
Psychotic symptoms (n = 5)	Primary psychosis, with differential diagnosis of developing dementia *	Medical treatment and psychotherapy
	Hallucinations due to medication (corticosteroids and morphine)	Medical treatment, advise reduce medication dose
	Severe depressive disorder with psychotic characteristics and suicidality, differential diagnosis side effects of levetiracetam	Hospitalization with medical treatment and psychotherapy, advise reduce levetiracetam dose
	Hallucinations and memory problems caused by GBM location and infiltration	Medical treatment
	Patient referred, but deteriorated quickly before evaluation could take place	–
Mood symptoms (n = 6)	Suicidal expressions with death wish but no active suicidality	Medical treatment
	Depressive symptoms, but no depressive disorder. Differential diagnosis side effects of levetiracetam	No specific psychiatric treatment, advise reduce levetiracetam dose
	Vital depressive disorder (severe) #	Medical treatment
	Depressive disorder (moderate/severe)	Medical treatment
	Depressive disorder due to somatic condition	Medical treatment and psychotherapy
	Patient referred, but lost to follow up for unknown reasons	-
Symptoms of anxiety (n = 3)	Anxiety disorder with depressive characteristics #	Medical treatment and psychotherapy
	Derealisation due to anxiety and mild depressive complaints. Differential diagnosis side effect of levetiracetam or corticosteroids	Psychotherapy, advise reduce levetiracetam and corticosteroid dose
	Somatic fixation/obsession stool pattern with depressive symptoms	Medical treatment
Behavioral symptoms or changed habit (n = 9)	Psycho-organc syndrome with behavioral disorder	Medical treatment
	Behavioral changes, no mania	No specific psychiatric treatment
	Disinhibition due to corticosteroid use	Medical treatment, advise reduce corticosteroid dose
	Coping problems, no psychiatric disorder	Psychotherapy
	Agitation and aggression due to misunderstanding, differential diagnosis due to corticosteroids	Medical treatment, advise reduce corticosteroid dose
	Coping problems, no psychiatric disorder	Psychotherapy
	Behavioral changes induced by radiotherapy, no psychiatric disorder	Medical treatment
	Aggression, delusions and hallucinations due to levetiracetam. Differential diagnosis primary psychosis #	Medical treatment
	Patient referred, but deteriorated quickly before evaluation could take place	–
Somatic symptoms, medical unexplained (n = 1)	Sleeping problems, no signs of anxiety or depression	Medical treatment and psychotherapy

*Temporarily interrupted their treatment due to symptoms

^#^Permanently discontinued their treatment due to symptoms

### Comparison between referred and non-referred patients

**Fig. 2:**
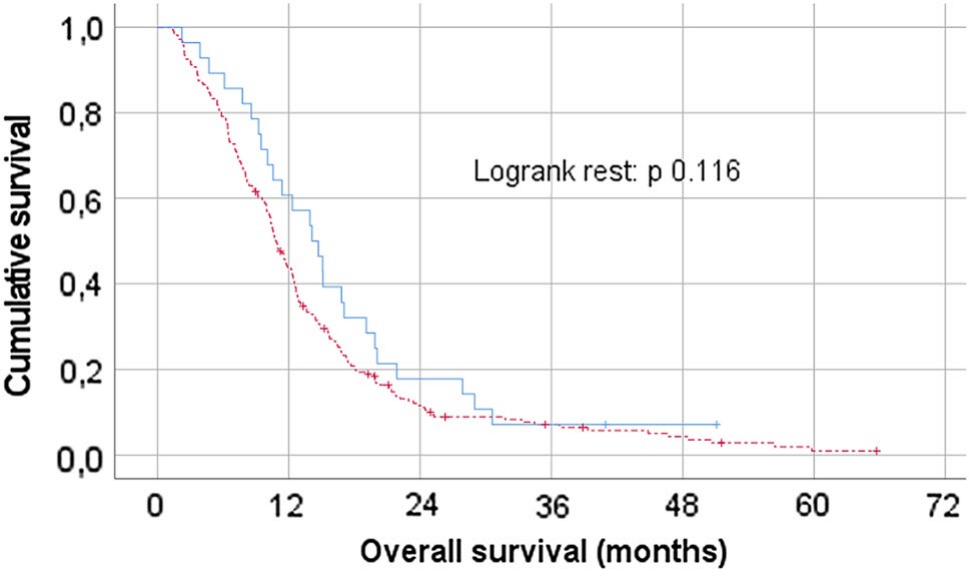
Kaplan–Meier overall survival curves comparing treated GBM patients with clinically relevant psychiatric symptoms (blue/continuous line) and patients without (red/dashed line)

Characteristics of both groups are displayed in Table 3*.* Referred patients were significantly more likely to be male, have a non-frontal tumor location and had more frequently a history of a psychiatric disorder. Furthermore, referred patients used more often anti-epileptic drugs at baseline and developed a postoperative delirium more frequently compared to non-referred patients. In addition, no use of corticosteroids at baseline was correlated with an increased risk of referral throughout oncological treatment or active surveillance. A Kaplan–Meier analysis did not reached significance (log rank *p* = 0.116), in an exploratory overall survival comparison (Fig. 2).

**Table 3:** Comparison between treated patients referred to a psychiatry and treated patient not referred

Variables	Referred (n = 28)	Non-referred (n = 216)	P-value
Gender, n (%) –Female –Male	5 (17.9) 23 (82.1)	81 (37.5) 135 (62.5)	**0.041**
Age at diagnosis, years –Mean (SD) –Median (range)	60.2 (11.7) 62.5 (33.5–79.4)	63.9 (10.1) 65.2 (26.0–84.6)	0.102
Type of surgery, n (%) (Partial) resection –Biopsy	19 (67.9) 9 (32.1)	120 (55.6) 96 (44.4)	0.216
Tumor location, n (%) –Left hemisphere –Right hemisphere	16 (57.1) 12 (42.9)	95 (44.0) 110 (50.9)	0.442
Tumor location, n (%) –Frontal –Non-frontal	3 (10.7) 25 (89.3)	82 (38.0) 134 (62.0)	**0.004**
Tumor location, n (%) –Temporal –Non-temporal	16 (57.1) 12 (42.9)	101 (46.8) 115 (53.2)	0.301
Chemoradiation + adjuvant chemotherapy as first treatment (Stupp/Elderly*), n (%) –Yes –No	25 (89.3) 3 (10.7)	166 (76.9) 50 (23.1)	0.133
ECOG score at baseline, n (%) –0 or 1 – ≥ 2	24 (85.7) 4 (14.3)	170 (78.7) 46 (21.3)	0.387
History of psychiatric disorder, n (%) –Yes -No	5 (17.9) 23 (82.1)	13 (6.0) 203 (94.0)	**0.041**
Use of antipsychotic medication at baseline, n (%) –Yes –No	2 (7.1) 26 (92.9)	18 (8.3) 198 (91.7)	1.000
Use of dexamethasone at baseline, n (%) –Yes –No	13 (46.4) 15 (53.6)	158 (73.1) 58 (26.9)	**0.004**
Use of anti-epileptic medication at baseline, n (%)* –Yes –No	15 (53.6)* 13 (46.4)	70 (32.4)** 146 (67.6)	**0.027**
Cognitive and/or behavioral symptoms at first presentation, n (%) –Yes –No	1 (3.6) 27 (96.4)	38 (17.6) 178 (82.4)	0.058
Postoperative delirium, n (%) –Yes –No	4 (14.3) 24 (85.7)	8 (3.7) 208 (96.3)	**0.036**

Bold font indicates statistical significance with a* P*-value of <0.05

N = number; SD = standard deviation; STUPP/ Elderly = radiotherapy plus concomitant and adjuvant Temozolomide

*14 patients used levetiracetam and 1 patient used levetiracetam and lacosamide

**59 patients used levetiracetam, 1 carbamazepine, 4 valproic acid, 2 lamotrigine, 1 lacosamide, 1 primidon and 1 levetiracetam + valproic acid

## Discussion

To our knowledge, this study is the first cohort-investigation to estimate the overall proportion of clinically relevant psychiatric comorbidity in GBM patients. The current study shows that 11.5% of patients developed clinically relevant psychiatric symptoms during GBM treatment or active surveillance. Most referrals concerned mood symptoms, behavioral disturbances or changed habit and psychotic symptoms. About two-third of all referrals took place during first line treatment with concomitant chemoradiation and adjuvant temozolomide. Because of the detrimental effect of psychiatric disturbances, 17.9% of patients discontinued their glioblastoma treatment and 3.6% temporarily interrupted their treatment.

Our findings are in line with literature on brain tumors in general and inform the field further. Although neurobehavioral symptoms are common in patient with brain tumors, reported prevalence vary widely. Changes in personality or behavior in glioma patients are reported ranging from 8 to 67% [14], depression occurs in 15% and anxiety in up to 25% [15, 16], however the prevalence of psychosis is yet unknown. [5] In a large prospective study investigating patterns of care for adults with newly diagnosed glioma, 1.8% of GBM patients were using antipsychotic medication during the perioperative period, although no long-term antipsychotic medication use was assessed. [17] Some studies have investigated the incidence of psychiatric symptoms in brain tumors in general at time of diagnosis. [7–13] They reported incidences ranging from 50 to 90%, most frequently mood symptoms. These results are comparable to symptoms that are reported by patients and caregivers in the doctor’s office during treatment and active surveillance in our study, but more importantly, our study revealed 11.5% of clinically relevant psychiatric symptoms. This difference is likely due to the difference in definition, in which we attempted to include some degree of severity and clinical relevance. For future studies, a consensus in the staging and grading of psychiatric symptoms and disorders is required, which will enable a better comparison across studies and patient populations. In addition, brain tumors in general or different glioma grades are often analysed altogether [3, 5, 7], making it difficult to draw firm conclusions on specific tumor types, such as GBM. Currently, risk factors for the development of psychiatric symptoms are largely lacking. In our study, we found that males, patients using anti-epileptic drugs at diagnosis and patients with a history of a psychiatric disorder were more at risk to develop psychiatric symptoms during glioblastoma treatment or active surveillance. In addition, patients who developed a postoperative delirium after glioblastoma surgery also developed more frequently psychiatric disturbances later on. In our study 6.3% developed a delirium, which is comparable to the study reported by Flanigan et al. [18] (7%).

We found that patients with a non-frontal glioblastoma location were more prone to develop psychiatric symptoms compared to a frontal glioblastoma. In general, it is thought that psychiatric symptoms may not have any localizing value. [6] However, there is anecdotal evidence that brain tumors in the limbic system, such as the amygdala, might be associated with psychotic symptoms [19, 20] and mood symptoms such as depression with frontal tumors [6]. One explanation for the found association in our study between psychiatric symptoms and non-frontal tumors could be that we excluded patients whit psychiatric symptoms at diagnosis. It may be that patients with a frontal tumor already had psychiatric symptoms at baseline, excluding them from the cohort of patients developing clinically relevant symptoms during oncologic treatment. Therefore, our finding that a non-frontal tumor location is associated with psychiatric symptoms should be interpreted with caution, and validation of our findings is needed in prospective studies, which may also enable possibilities to unravel underlying mechanisms. Our results may suggest that certain patient categories, e.g., patients with a psychiatric history or patients on certain medication with a non-frontal tumor, may benefit from specific education or stringent monitoring by a psychologist and psychiatrist during the treatment process.

A very important finding is that according to the evaluating psychiatrists, up to one third of the clinically relevant psychiatric symptoms were triggered by medication, mainly corticosteroids and anti-epileptic drugs. According to Warrington & Bostwick [21] the use of corticosteroids can lead to severe psychiatric side effects in 6% of patients and mild to moderate side effects in about 28%. We postulate that especially initiation of corticosteroid use is correlated with the onset of psychiatric disturbances, which is substantiated by the finding that no corticosteroid use at baseline was correlated with an increased risk of psychiatric referral throughout the disease course and additionally by the fact that up to 80% of referred patients were using corticosteroids at time of psychiatric consultation. Beside monitoring of specific patient groups more closely, our findings also emphasize the importance of drug monitoring during treatment or active surveillance, with mandatory selection of patients who benefit and who don’t benefit from symptomatic treatments.

Next to clinically relevant psychiatric symptoms, a variety of milder symptoms were discussed during outpatient clinic visits. Although 91% of treated patients reported at least one symptom, mostly fatigue, cognitive or behavioral problems, only a small portion were ultimately referred to a psychiatrist in our study. In our study, the referral exception was due to psychotic symptoms, in which 17.9% of patients were referred. Our findings are in accordance with Boele et al. [22], who reported that symptoms of fatigue, cognitive deficits, depression and changes in personality and behavior are frequently reported in glioma patients and have a large impact on the everyday life of patients and their partners. The reason for the fact that only a minority of patients reporting symptoms was eventually referred to a psychiatrist is still unclear. One explanation could be that a referral is considered as extra patient burden, with already frequent hospital visits during their treatment cause. Another explanation could be that only patients with severe symptoms were referred to a psychiatrist, and milder symptoms were initially managed by the treating physician or general practitioner themselves. It is important that future studies use uniform criteria and validated screening or measurement tools to specifically address psychiatric symptoms in glioma patients. Patient and caregiver reported outcomes (PROs) or measurements (PROMs) are available for glioblastoma patients, such as the EORTC QLQ-30, but these are not specific for psychiatric disturbances, with only a few questions addressing these items. Other tests, such as the neuropsychiatric inventory (NPI) could be more informative in that case. Ideally, if specific risk factors will be identified in future prospective studies, a more specific risk screening tool should be developed to identify and monitor specific patients at risk for psychiatric disturbances. Another issue is the timing of standardized measurements. Guidelines advise routine screening at time of diagnosis and then every six months, with, if necessary, referral to mental health specialists for further evaluation and interventions. [23] Since, patients in our study frequently developed psychiatric symptoms between baseline and 6 months after diagnosis, it could be beneficial to specifically train health care providers to recognise specific psychiatric symptoms during early oncologic treatment. Early recognition may not only improve patients functioning and QoL [24–26], but might also prevent discontinuation of care or treatment refractoriness. In addition, given that relevant but undetected, and therefore, untreated affective symptoms cause higher care utilization, an integrated multidisciplinary care trajectory could be recommended in complex cases. Identification of such cases at baseline should be one of the primary aims in future studies [27].

Although we did not find a significant difference in overall survival between patients treated and not-treated for psychiatric disturbances, there is presumptive evidence that psychiatric or psychologic treatment or counselling can have a positive effect on survival. Berchuck et al*.* [28] found a significant improvement in cancer mortality in Veterans with non-small cell lung cancer and mental health disorders who participated in support programs and Fu et al*.* [29] found that psychosocial interventions demonstrated improvements in survival in patients with cancer. These findings are in contrast to the effect of cognitive impairment, which is a known negative prognostic factor in glioma patients [30, 31]. Although the exact underlying mechanism is unknown, van Kessel et al. [31] postulate that cognition is (a) a marker for diffuse tumor infiltration, (b) cognition and survival might share specific (genetic) risk factors and (c) cognition influences treatment decision making and treatment compliance, all negatively effecting survival. Although our exploratory results are preliminary and speculative, the seemingly opposite effects of primary psychiatric symptoms in comparison to cognitive symptoms on survival might point to distinct underlying mechanisms and risk factors. Further studies are warranted to investigate and unravel these risk factors and possible mechanisms, with a reported incidence of 11.5% in our study, justifying more research.

Our study has limitations. First, we chose to define “clinically relevant” as psychiatric symptoms that required a referral to a psychiatrist, which likely induced underreporting. Given the proportion of undetected psychiatric symptoms in complex clinical cohorts, this is probably a definition with referral bias. Consecutively, less severe symptoms may be not reported and could be untreated or managed by the treating physicians themselves, and, thus, (falsely) deemed not severe enough to initiate a referral. Alternatively, non-psychiatric symptoms could have been more prominent, such as, progressive neurologic deterioration, deciding that a psychiatric consultation had no priority. Therefore, our reported incidence is probably an underestimation, but already justifies more research attention. The current lack of uniform criteria and validated screening or measurement tools should be adequately addressed in future studies. Second, our study relied on the judgement of the treating physician, and some doctors may refer patients faster than others or make more extensive notes than others, which introduced further selection and information bias. However, due to the long inclusion period and treatment by several specialists during the treatment and follow up period, we think that our findings are representative for routine neuro-oncology in daily practice. Third, only information from files in MUMC + and ZMC could be included in the study, no additional information from external psychiatrists could be retrieved. Last but not least this study has a retrospective design with its associated restrictions.

## Conclusions

Our study shows that 11.5% of newly diagnosed GBM patients developed clinically relevant psychiatric symptoms, mainly during concomitant chemoradiation or adjuvant chemotherapy. Because of their detrimental effect, 17.9% of patients with psychiatric symptoms discontinued their treatment and 3.6% temporarily interrupted their treatment. Although, up to 28.6% of the clinically relevant psychiatric symptoms were thought to be attributable to medication, further prospective studies are needed to identify specific risk factors for and possible effects of psychiatric comorbidity on overall survival. The reported incidence and the high number of patients discontinuing their treatment due to psychiatric symptoms justifies more research in GBM. To this date, GBM with psychiatric comorbidity is an understudied topic in the current scientific literature.

## Supplementary Information

Below is the link to the electronic supplementary material.

Supplementary file1 (DOCX 412 KB)

## Data Availability

The data that support the findings of this study are available from the corresponding author, [MPGB], upon reasonable request.
