# Supplementary material for: Incidence of clinically relevant psychiatric symptoms during glioblastoma treatment: an exploratory study
Source: J Neurooncol. 2023 May 10;163(1):185–94. doi: 10.1007/s11060-023-04326-2 (PMC10232638; doi:10.1007/s11060-023-04326-2)
Supplement: Supplementary file 1 — Supplementary file1 (DOCX 412 KB) [file 11060_2023_4326_MOESM1_ESM.docx]

**Supplementary**

| Supplementary table 1. Patient characteristics treated versus not treated | | | | |
| --- | --- | --- | --- | --- |
| Variables | **Treated (n=244)** | **Not treated (n=53)** | **P-value** |  |
| Gender, n (%)   - Female - Male | 86 (35.2) 158 (64.8) | 22 (41.5) 31 (58.5) | 0.390 |  |
| Age at diagnoses, years   - Mean (SD) - Median (range) | 63.4 (10.3) 64.9 (26.0-84.6) | 69.0 (8.3) 68.5 (52.5-88.0) | **<0.001** |  |
| Type of surgery, n (%)   - (Partial) resection - Biopsy | 139 (57.0) 105 (43.0) | 7 (13.2) 46 (86.8) | **< 0.001** |  |
| MGMT hypermethylation, n (%)   - Yes - No | 98 (40.2) 146 (59.8) | 25 (47.2) 28 (52.8) | 0.348 |  |
| ECOG score at baseline, n (%)   - 0 or 1 - ≥ 2 | 194 (79.5) 50 (20.5) | 15 (28.3) 38 (71.7) | **< 0.001** |  |
| Use of dexamethasone at baseline, n (%)   - Yes - No | 171 (70.1) 73 (29.9) | 33 (62.3) 20 (37.7) | 0.266 |  |
| Overall survival, months   - Mean (SD) | 13.6 (10.8) | 1.9 (1.6) | **<0.001** |  |

n, number; SD, standard deviation; MGMT, 06-methylguanine-DNA-methyltransferase; ECOG, Eastern Cooperative Oncology Group Performance Status

| Supplementary table. 2 Descriptive symptoms classified into distinct categories |
| --- |
| Cognitive symptoms   - Memory problems - Concentration problems - Signs of disorientation - Slowed thinking and processing of information - Difficulty with motor planning to perform tasks or to finish tasks - Diminished awareness of illness - Signs of confusion (not otherwise specified) |
| Psychotic symptoms   - Visual hallucinations - Acoustic hallucinations - Paranoid and/or delusional thoughts - Symptoms of derealisation |
| Mood symptoms   - Lack of interest or enthusiasm - Concern or excessive worrying - Being unusually or excessively emotional - Feeling down - Suicidal thoughts/expressions |
| Symptoms of anxiety   - Anxiety - Panic attacks - Obsession or pre-occupation with someone or something |
| Behavioral symptoms or changed habit   - Symptoms of inner unrest or uneasiness - Deterioration in public decorum - Signs of aggression - Signs of disinhibited behavior - Lack of initiative - Behavioral changes (not otherwise specified) - Coping problems - Shows less/more interest in sex |
| Somatic symptoms   - Fatigue - Sleeping problems - Decreased appetite or involuntary weight loss |

**Supplementary figure 1.** Overview of reported psychiatric/psychological symptoms during at least one outpatient clinic visit


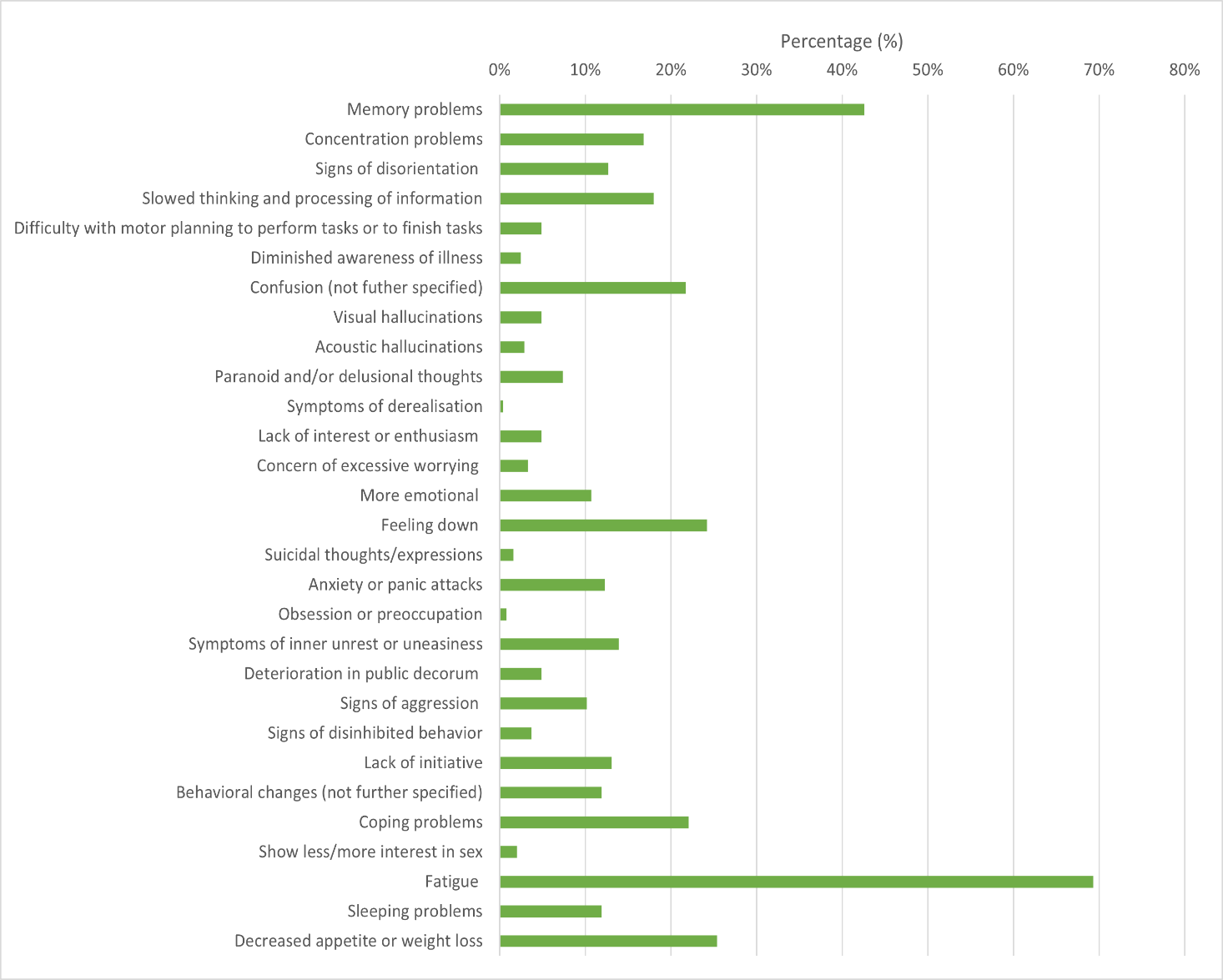


The wide variety of descriptive symptoms were classified into 6 categories: cognitive symptoms, psychotic symptoms, mood symptoms, symptoms of anxiety, behavioral symptoms or changed habit and somatic symptoms (see *supplementary* *table 2*). They were present at some point during treatment or active surveillance in 64.8%, 11.5%, 34%, 12.7%, 48.8% and 75% of patients, respectively.
